# The burden of low back pain and predictions in Asia–Pacific region, 1990–2021: a comparative analysis of China, Japan, Thailand, and Pakistan

**DOI:** 10.3389/fmed.2026.1693067

**Published:** 2026-02-04

**Authors:** Chaoshun Zheng, Haojia Huang, Longsheng Zhang, Yanzhen Wang, Jiabin Li, Yueyue Guo, Xuhui He

**Affiliations:** 1 Department of Orthopedics II, Jieyang People’s Hospital, Jieyang, China; 2 Department of Neurology, Jieyang People’s Hospital, Jieyang, China; 3 Department of Anesthesiology, Jieyang People’s Hospital, Jieyang, China; 4 Department of Endocrinology, Jieyang People’s Hospital, Jieyang, China; 5 Radiotherapy Department, Jieyang People’s Hospital, Jieyang, China

**Keywords:** age-period-cohort modeling, Asia–Pacific, disability, epidemiology, low back pain, public health policy, temporal trends

## Abstract

**Objective:**

Low back pain (LBP) is a leading cause of disease burden, imposing substantial societal costs. However, compared to infectious diseases, it receives insufficient attention in the Asia-Pacific region, with limited research and resource allocation. This study examines the characteristics of LBP disease burden in representative Asia-Pacific countries—China, Japan, Thailand, and Pakistan—from 1990 to 2021, and projects trends to 2050, aiming to inform evidence-based health policies.

**Methods:**

Utilizing Global Burden of Disease (GBD) 2021 data, we analyzed LBP incidence, prevalence, and years lived with disability (YLDs). Country comparisons employed age-standardized rates (ASRs), stratified by age groups. Joinpoint regression assessed trends during 1990–2021 and compared cross-national variations. The BAPC model was used to predict future LBP disease burdens from 2022 to 2050. Decomposition analysis quantified contributions from population aging, population growth, and epidemiological changes.

**Results:**

In 2021, Japan exhibited the highest ASRs, followed by China, Thailand, and Pakistan. Case counts peaked in the working-age population, while ASR peaks concentrated in middle-aged and older adult groups. Females consistently bore a higher burden than males. From 1990 to 2021, China and Japan showed declining ASR trends, whereas Thailand and Pakistan demonstrated upward ASR trends; all countries saw rising case counts. BAPC projections indicated increasing ASR burdens for Chinese males and Japanese females by 2050, with declines in other groups. Decomposition analysis revealed divergent drivers across countries.

**Conclusion:**

LBP remains a major public health challenge in these representative Asia-Pacific countries, with age, gender, and national heterogeneity shaping burden dynamics. Tailored policies addressing country-specific, gender-specific, and age-specific disparities are urgently needed to mitigate the disease burden of LBP.

## Introduction

Low back pain (LBP) is an extremely common symptom, defined as pain in the back between the lower ribs and the groin, and serves as a significant cause of disability across nearly all age groups in countries of varying income levels, leading to activity limitation and work absence (1). Approximately 90% of lower back pain cases are idiopathic, with only 5–10% attributable to identifiable causes such as vertebral fractures, tumors, infections, lumbar disk herniation, etc. (2). Driven by population growth, aging, greater occupational demands, obesity, and other factors, lower back pain has progressively emerged as a major global health challenge, with the 1-year incidence of a first-ever episode of LBP ranging from 6.3 to 15.4% and the median 1-year period prevalence among adults is 37% globally (3, 4).

In fact, low back pain affects individuals across nearly all age groups and spans high-, middle-, and low-income populations (1). According to the 2021 GBD study, low back pain ranked as the leading cause of years lived with disability (YLDs) in 2021, accounting for 7.7% of all-cause YLDs with an age-standardized rate of 832.2 per 100,000 population (5). Moreover, the costs, healthcare utilization, and disability associated with low back pain are heavily influenced by local cultural, social, and health systems, resulting in substantial variations across countries. Based on GBD 2021 data, the age-standardized rate of disability-adjusted life years (DALYs) due to low back pain was highest in high-SDI countries (1094.3 per 100,000) and lowest in middle-SDI countries (717.5 per 100,000) (5). However, in low- and middle-income countries—particularly in Asia, Africa, and the Middle East—the burden of low back pain is projected to continue rising over the coming decades (1). The health and social systems in these regions may currently be insufficient to address the impending challenges posed by the growing burden of low back pain. The economic burden of low back pain cannot be overlooked, imposing heavy costs on individuals, health systems, and society—comparable to high-cost epidemics like cardiovascular diseases, cancer, and mental health disorders (6). However, global resource allocation remains disproportionately low, particularly in Asian countries where investments are often prioritized toward infectious disease prevention and treatment (7). Previous GBD studies indicate a weak positive correlation between low back pain burden and Socio-demographic Index (SDI) levels, with prevalence rates of 32.9, 25.4, and 16.7% in high, middle and low-SDI countries, respectively (4). Yet, existing research predominantly focuses on individual nations or global aggregates, leaving a gap in GBD-specific studies for the Asia-Pacific region. Projections indicate that by 2050, the total number of low back pain cases will increase by 36.4%, with the most substantial rises expected in Asia and Africa (5). Investigating the characteristics of low back pain burden in representative Asia-Pacific countries (across varying SDI levels) will facilitate the formulation of targeted health policies, ultimately alleviating the associated societal burden in diverse Asian contexts.

This study examines the characteristics of low back pain burden—including incidence, prevalence, and YLDs—in four Asia-Pacific countries (China, Japan, Thailand, Pakistan) from 1990 to 2021, while projecting trends for 2022–2050. The analysis encompasses disparities across age groups, genders, and nations. A decomposition analysis quantifies the contributions of drivers such as population growth, aging, and epidemiological changes. Findings aim to provide a data foundation for evidence-based formulation of targeted healthcare policies.

## Methods

### Data source and disease definition

The GBD 2021 integrates epidemiological data from 204 countries and regions over the period of 1990–2021 (8). Data were primarily obtained through the extensive GBD Collaborator Network and are subject to strict quality assessment (8). The main statistical results include incidence, prevalence, mortality, and DALYs for 371 diseases or injuries, including low back pain (8). Low back pain is also a leading Level 3 cause of YLDs (8). According to the official GBD definition (9), low back pain is defined as pain in the area on the posterior aspect of the body from the lower margin of the twelfth ribs to the lower gluteal folds (with or without pain referred into one or both lower limbs) that lasts for at least 1 day.

After data collection, potential biases in the dataset were rigorously assessed and adjusted. The prevalence and incidence of low back pain were modeled using DisMod-MR 2.1 (8), which is a Bayesian disease modeling meta-regression tool. This method allows for data analysis by sex, location, year, and age group. Crude YLD rates were assessed by multiplying multiple sequela-specific prevalence by their respective disability weights (8). This study focused on the incidence, prevalence, and YLD burden of low back pain. The study included four countries in the Asia-Pacific region and demonstrated the disease burden of low back pain in the region through different SDI levels, providing data for relevant medical decision-making in the Asia-Pacific region.

It is important to note that the GBD 2021 model accounts for uncertainty arising from multiple sources, such as sampling error, measurement error, and model specification, which is especially critical for countries with data-sparse or suboptimal data quality.

### Burden description

In this study, we used annual incident cases, prevalent cases, YLDs, and the corresponding age-standardized rates to illustrate the burden of low back pain. The data were stratified by country and sex. After standardization, the age-standardized rates (ASRs) can be compared across different countries. The 95% uncertainty intervals (UIs) were determined by the 25th and 975th estimates of the 1,000 runs after ordering from smallest to largest (10, 11).

### Trends analysis

To explore the change trends of lower back pain from 1990 to 2021, we used the join-point regression model for analysis. This model employs the least squares approach, which can avoid subjectivity in traditional trend analysis and identify trend inflection points (12). To quantify the degree of trend change, we calculated the average annual percentage change (AAPC) and the annual percentage change for each segment, both expressed in the form of 95% confidence intervals (CIs). The *p*-value, determined by comparing the annual percentage change with 0, indicates whether the trend is statistically significant. A *p*-value less than 0.05 is considered statistically significant.

### Bayesian age-period-cohort analysis

The BAPC model, compared to traditional models, can separately analyze the independent effects of age, period, and cohort, solving the collinearity problem among the three and predicting the trend of disease burden. In this study, by analyzing the four selected countries, we obtained the impact of different factors on low back pain in countries with different SDI levels, providing highly credible evidence for the formulation of more precise public health decisions. In our BAPC model, we employed the GBD world population age standard from the 2024 Lancet publication as the reference age structure (13). Through this model, we also predicted the disease burden of low back pain from 2022 to 2050.

### Decomposition method

By employing the decomposition analysis method to calculate the contribution of population growth, aging, and epidemiological factors to the trend of the burden of low back pain, it is possible to intervene in the factors with significant contributions to achieve the goal of precisely reducing the disease burden. In this study, we employed the Das Gupta decomposition method (14) to analyze the number of cases of incidence, prevalence, and YLDs for each country. Taking YLDs as an example, the total YLDs for a region in a given year y was calculated using the following formula: $\mathrm{YLD}s_{a_{y},p_{y},e_{y}}=\sum_{i=1}^{20}(a_{i,y}\times p_{y}\times e_{i,y})$ . $\mathrm{YLD}s_{a_{y},p_{y},e_{y}}$ represents the YLDs based on the age structure, population size, and YLDs rate for a specific year *y*; 𝑎_𝑖,𝑦_ represents the proportion of the population in age group *i* out of 20 age groups in year *y*; 𝑝_𝑦_ represents the total population in year *y*; and 𝑒_𝑖,𝑦_ represents the YLDs number in age group *i* in year *y*. By isolating other variables, we quantified the unique impact of a single factor on the changes in YLDs.

In countries with different SDI levels, conducting decomposition analysis to determine the contribution of different factors to the disease burden can also enable the formulation of targeted public health policies.

### Selection of study countries in the Asia-Pacific region

Given the large number of countries in the Asia-Pacific region, this study selected a limited yet representative set of nations to illustrate key patterns. The selection was primarily based on covering a range of Socio-demographic Index (SDI) levels, geographic locations, and large populations to enhance representativeness: Japan (High SDI), serving as a benchmark for developed, aging societies; China (Middle-high SDI), representing a rapidly developing and populous nation; Thailand (Middle SDI), exemplifying a major middle-income economy in Southeast Asia; and Pakistan (Low-middle SDI), reflecting a populous lower-middle-income country in South Asia. This gradient in development levels allows the analysis to examine how low back pain (LBP) burden patterns may vary across stages of socioeconomic development. Moreover, these countries capture diverse cultural, occupational, and lifestyle contexts that influence LBP risk factors, such as aging populations in Japan, agricultural labor in Thailand and parts of China, and rapid urbanization in China and Pakistan. The four countries also differ markedly in health system maturity, stage of epidemiological transition, and potential policy entry points for LBP management—ranging from advanced public health infrastructure in Japan to health systems still prioritizing infectious disease control in Pakistan. These contrasts offer practical insights for tailoring prevention and management strategies across different development contexts.

All analyses and visualizations in this paper were conducted using the R language (version 4.4.1), and a *p*-value less than 0.05 was considered to indicate a statistically significant difference.

## Results

### Burden of low back pain among China, Japan, and Thailand, and Pakistan in 2021

Globally, low back pain caused 266,873,321 (95% UI: 235369489–299,406,380) incident cases, 628,838,475 (95% UI: 551834407–700,881,341) prevalent cases, and 70,156,962 (95% UI: 50194205–94,104,688) YLDs in 2021. The age-standardized incidence rate (ASIR), prevalence rate (ASPR), and YLD rate (ASYR) were 3176.63 (95% UI: 2811.82–3562.29), 7463.13 (95% UI: 6575.68–8321.8), 832.18 (95% UI: 595.85–1115.24) per 100,000 population, respectively. In China, low back pain caused 43,374,995 (95% UI: 37494376–49,159,184) incident cases, 100,093,746 (95% UI: 87128173–113,014,316) prevalent cases, and 11,297,805 (95% UI: 7931468–15,328,056) YLDs in 2021. The age-standardized incidence rate (ASIR), prevalence rate (ASPR), and YLD rate (ASYR) were 2342.46 (95% UI: 2058.05–2639.32), 5342.1 (95% UI: 4660.41–5976.28), 603.03 (95% UI: 427.63–810.16) per 100,000 population, respectively. In Japan, low back pain caused 8,085,995 (95% UI: 7116421–9,043,618) incident cases, 20,008,724 (95% UI: 17610370–22,194,703) prevalent cases, and 2,232,624 (95% UI: 1590996–2,995,120) YLDs in 2021. The ASIR, ASPR, and ASYR were 4447.66 (95% UI: 3939.3–5022.53), 10626.15 (95% UI: 9373.76–11935.56), 1208.31 (95% UI: 861.08–1627.71) per 100,000 population, respectively. In Thailand, low back pain caused 2,075,125 (95% UI: 1796308–2,348,951) incident cases, 4,771,838 (95% UI: 4142456–5,430,805) prevalent cases, and 534,708 (95% UI: 377829–729,711) YLDs in 2021. The ASIR, ASPR, and ASYR were 2284.91 (95% UI: 1998.55–2581.55), 5169.73 (95%UI: 4487.19–5814.51), 581.13 (95% UI: 413.21–789.1) per 100,000 population, respectively. In Pakistan, low back pain caused 6,112,580 (95% UI: 5300163–7,034,456) incident cases, 13,925,090 (95% UI: 12007269–16,056,430) prevalent cases, and 1,555,725 (95% UI: 1091574–2,118,767) YLDs in 2021. The age-standardized incidence rate (ASIR), prevalence rate (ASPR), and YLD rate (ASYR) were 3334.44 (95% UI: 2893.5,3769.61), 7787.21 (95% UI: 6686.41,8936.8), 861 (95%UI: 604.05,1170.82) per 100,000 population, respectively (Table 1).

**Table 1:** Burden of low back pain among China, Japan, and Thailand, and Pakistan in 2021 and AAPC from 1990 to 2021.

Location	Sex	Incidence			Prevalence			YLD		
		Number	ASR	AAPC	Number	ASR	AAPC	Number	ASR	AAPC
Global	Both	266,873,321 (235,369,489,299,406,380)	3176.63 (2811.82, 3562.29)	−0.34 (−0.37, −0.32)	628,838,475 (551,834,407,700,881,341)	7463.13 (6575.68, 8321.8)	−0.38 (−0.40, −0.35)	70,156,962 (50,194,205,94,104,688)	832.18 (595.85,1115.24)	−0.38 (−0.40, −0.36)
	Female	166,097,403 (146,916,668,186,192,888)	3879.94 (3438.63, 4344.9)	−0.31 (−0.32, −0.29)	396,747,972 (348,340,471,442,511,662)	9212.46 (8122.92, 10285.14)	−0.35 (−0.37, −0.34)	43,934,955 (31,447,685,58,945,143)	1021.52 (732.39,1370.45)	−0.36 (−0.37, −0.34)
	Male	100,775,918 (88,485,948,113,863,884)	2450.55 (2161.03, 2758.87)	−0.39 (−0.43, −0.36)	232,090,503 (203,351,350,259,931,953)	5640.23 (4965.63,6300.61)	−0.40 (−0.44, −0.36)	26,222,007 (18,702,285,35,396,348)	635.48 (453.91,854.29)	−0.40 (−0.45, −0.35)
China	Both	43,374,995 (37,494,376,49,159,184)	2342.46 (2058.05, 2639.32)	−0.61 (−0.66, −0.57)	100,093,746 (87,128,173,113,014,316)	5342.1 (4660.41,5976.28)	−0.66 (−0.71, −0.61)	11,297,805 (7,931,468,15,328,056)	603.03 (427.63,810.16)	−0.68 (−0.73, −0.64)
	Female	26,216,943 (22,616,551,29,717,547)	2779.16 (2436.19, 3121.16)	−0.71 (−0.75, −0.67)	60,945,208 (52,941,431,68,505,911)	6381.38 (5567.92,7153.22)	−0.79 (−0.83, −0.75)	6,823,284 (4,775,816,9,218,929)	716.15 (506.26,959.84)	−0.79 (−0.84, −0.75)
	Male	17,158,052 (14,905,295,19,511,511)	1901.62 (1673.22, 2155.4)	−0.50 (−0.53, −0.46)	39,148,537 (33,850,361,44,326,367)	4282.3 (3759.62,4838.55)	−0.49 (−0.53, −0.46)	4,474,521 (3,156,562,6,067,442)	488.36 (346.52,657.58)	−0.50 (−0.54, −0.46)
Japan	Both	8,085,995 (7,116,421,9,043,618)	4447.66 (3939.3, 5022.53)	−0.28 (−0.36, −0.19)	20,008,724 (17,610,370,22,194,703)	10626.15 (9373.76,11935.56)	−0.30 (−0.36, −0.25)	2,232,624 (1,590,996,2,995,120)	1208.31 (861.08,1627.71)	−0.30 (−0.35, −0.24)
	Female	5,102,688 (4,484,968,5,697,634)	5487.07 (4864.03, 6206.15)	−0.20 (−0.30, −0.11)	12,840,046 (11,365,965,14,194,279)	13274.19 (11803.95,14962.29)	−0.25 (−0.34, −0.16)	1,425,900 (1,024,023,1,908,071)	1505.38 (1074.06,2017.46)	−0.24 (−0.33, −0.15)
	Male	2,983,307 (2,618,682,3,360,036)	3405.23 (2993.36, 3847.75)	−0.28 (−0.33, −0.22)	7,168,677 (6,252,749,8,002,861)	7954.42 (6949.76,8975.88)	−0.28 (−0.34, −0.22)	806,725 (566,847,1,090,857)	909.21 (646.98,1230.86)	−0.26 (−0.28, −0.24)
Thailand	Both	2,075,125 (1,796,308,2,348,951)	2284.91 (1998.55, 2581.55)	0.08 (0.07, 0.09)	4,771,838 (4,142,456,5,430,805)	5169.73 (4487.19,5814.51)	0.14 (0.12, 0.16)	534,708 (377,829,729,711)	581.13 (413.21,789.1)	0.16 (0.14, 0.18)
	Female	1,364,556 (1,175,756,1,548,623)	2824.68 (2464.18, 3,207)	0.10 (0.09, 0.11)	3,184,618 (2,754,562,3,616,546)	6474.9 (5612.14,7295.42)	0.16 (0.15, 0.17)	355,136 (250,136,481,464)	725.41 (514.66,975.89)	0.17 (0.16, 0.19)
	Male	710,569 (613,189,808,865)	1683.71 (1472.96, 1917.32)	0.04 (0.02, 0.07)	1,587,219 (1,370,558,1,816,027)	3704.67 (3246.22,4214.25)	0.09 (0.06, 0.12)	179,572 (126,468,246,072)	419.66 (298.26,561.63)	0.11 (0.08, 0.14)
Pakistan	Both	6,112,580 (5,300,163,7,034,456)	3334.44 (2893.5, 3769.61)	0.28 (0.26, 0.30)	13,925,090 (12,007,269,16,056,430)	7787.21 (6686.41,8936.8)	0.32 (0.27, 0.36)	1,555,725 (1,091,574,2,118,767)	861 (604.05,1170.82)	0.32 (0.29, 0.36)
	Female	4,121,941 (3,594,830,4,740,068)	4557.79 (3970.78, 5137.26)	0.18 (0.16, 0.19)	9,565,057 (8,255,290,11,050,643)	10883.43 (9362.13,12441.76)	0.20 (0.16, 0.23)	1,062,333 (741,848,1,458,425)	1195.75 (846.91,1623.98)	0.19 (0.15, 0.23)
	Male	1,990,639 (1,694,908,2,277,522)	2170.29 (1855.95, 2472.84)	0.31 (0.29, 0.34)	4,360,033 (3,700,443,5,102,144)	4848.62 (4110.6,5574.27)	0.36 (0.32, 0.39)	493,392 (349,303,664,500)	542.89 (385.42,733.11)	0.35 (0.31, 0.40)

**Figure 1:**
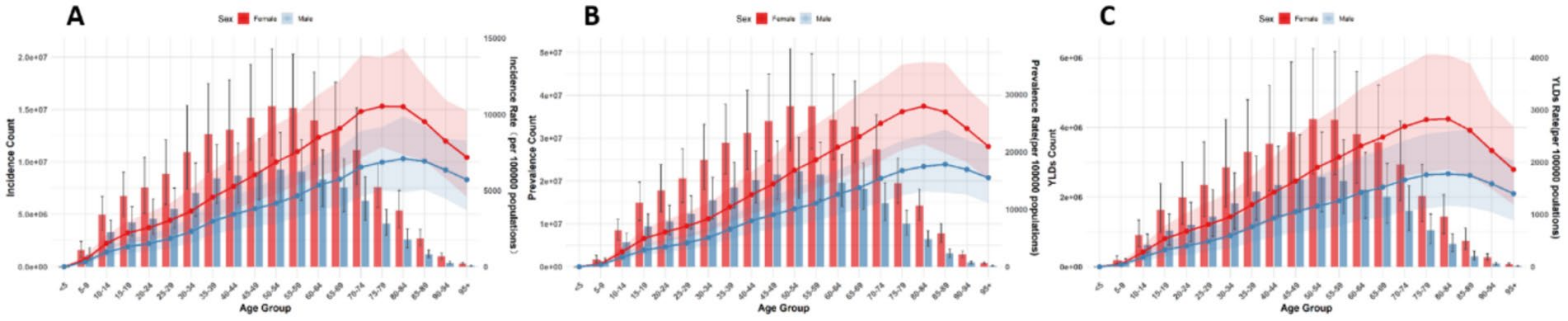
Burden of low back pain by sex and age globally in 2021. **(A)** Incidence, **(B)** Prevalence, **(C)** YLDs.

**Figure 2:**
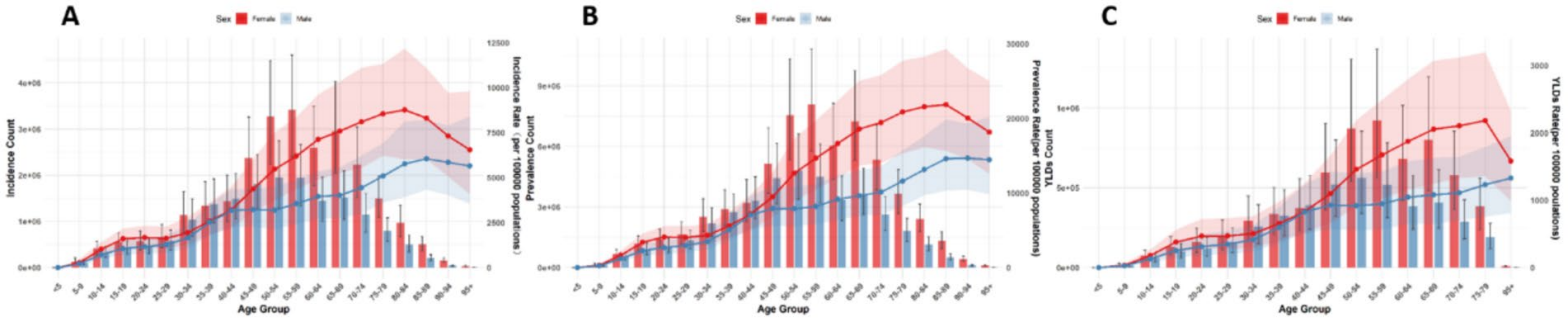
Burden of low back pain by sex and age in China in 2021: **(A)** Incidence, **(B)** prevalence, **(C)** YLDs.

**Figure 3:**
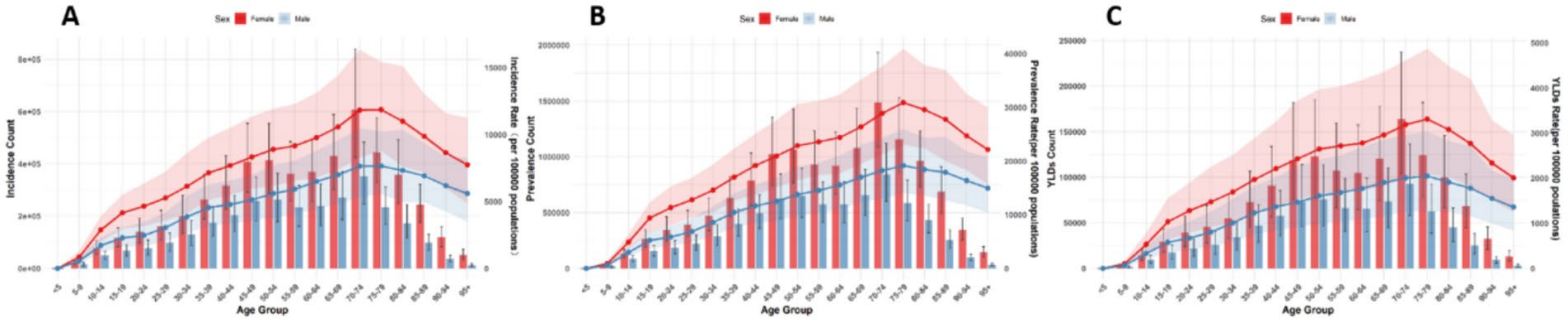
Burden of low back pain by sex and age in Japan in 2021. **(A)** Incidence, **(B)** Prevalence, **(C)** YLDs.

**Figure 4:**
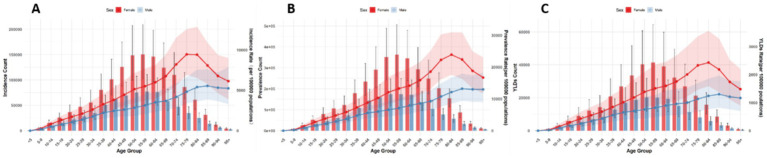
Burden of low back pain by sex and age in Thailand in 2021: **(A)** Incidence, **(B)** prevalence, **(C)** YLDs.

**Figure 5:**
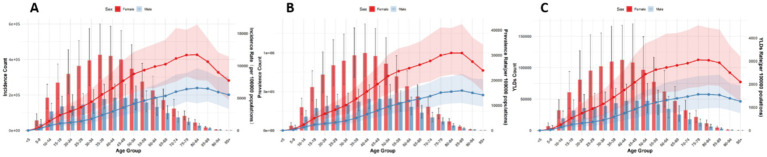
Burden of low back pain by sex and age in Pakistan in 2021: **(A)** Incidence, **(B)** Prevalence, **(C)** YLDs.

Globally, the number and ASRs of incidence, prevalence, and YLDs for low back pain exhibited a unimodal age pattern. The peak number occurred around 50–54 years globally and in China/Thailand, but shifted to 70–79 years in Japan and 35–44 years in Pakistan. The ASR peak was observed at 75–89 years globally and in China/Thailand/Pakistan, while Japan showed a concordant peak at 70–79 years. Notably, females bore a significantly higher burden than males across all age groups (Figures 1–5).

### Trends of low back pain among China, Japan, Thailand and Pakistan from 1990 to 2021

**Figure 6:**
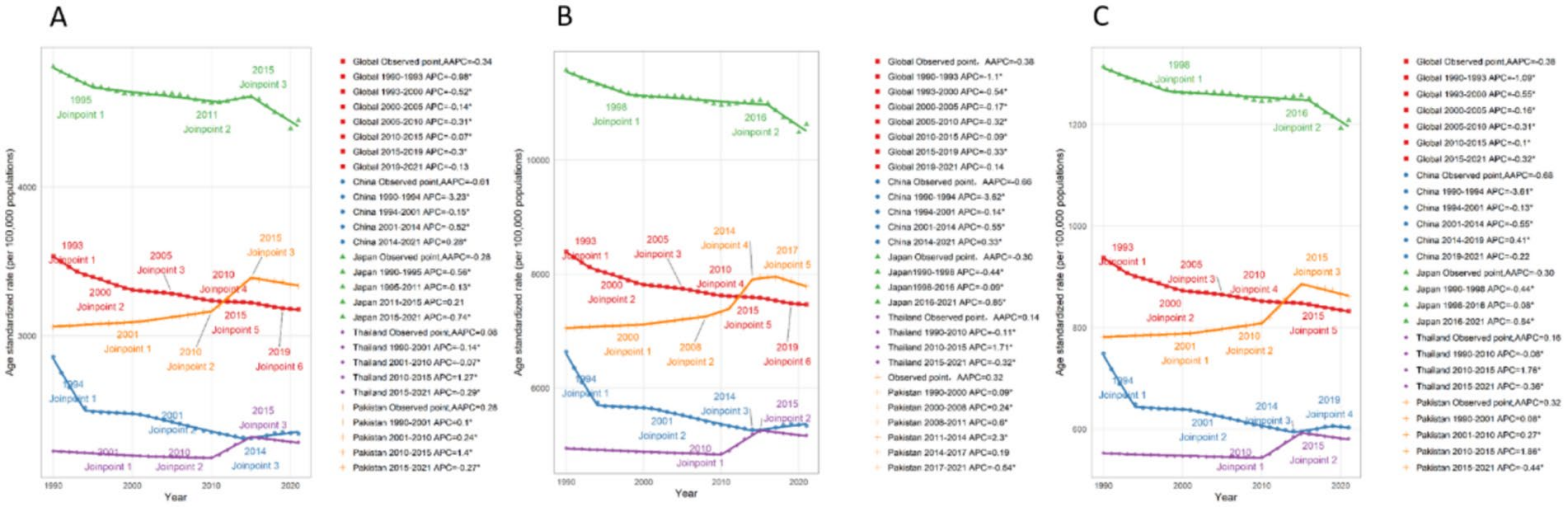
Average annual percent change among four countries from 1990 to 2021: **(A)** Incidence, **(B)** Prevalence, **(C)** YLDs.

**Figure 7:**
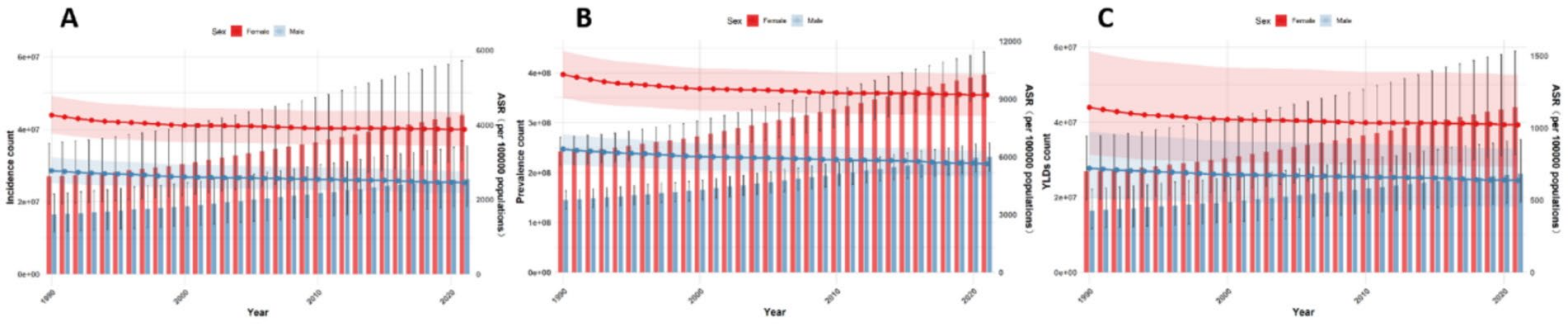
Number and rates of low back pain globally from 1990 to 2021 **(A–C)**. Incidence, prevalence, YLDs.

Between 1990 and 2021, the characteristics of the burden of low back pain varied among different countries. However, there were commonalities. In all countries included in this study, the burden among females was higher than that among males. At the global level, the rate of disease burden showed a stable downward trend during this period. The ASIR (Age-Standardized Incidence Rate), ASPR (Age-Standardized Prevalence Rate), and ASYR (Age-Standardized Years Lived with Disability Rate) were −0.34 (−0.37, −0.32), −0.38 (−0.40, −0.35), and −0.38 (−0.40, −0.36), respectively. The decline was more rapid between 1990 and 2000, and then tended to stabilize, with a downward trend observed in both males and females. However, in terms of absolute numbers, there was an upward trend. From 1990 to 2021, the number of incident cases, prevalent cases, and YLDs increased by 61.7, 62.6, and 61.7%, respectively (see Figures 6, 7).

**Figure 8:**
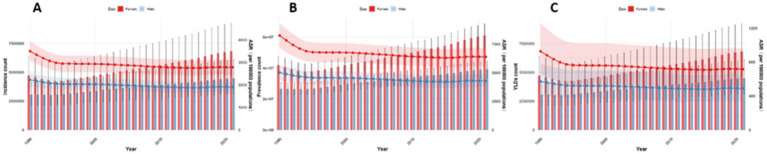
Number and rates of low back pain in China from 1990 to 2021. **(A)** Incidence, **(B)** prevalence, **(C)** YLDs.

In China, the disease burden of low back pain is on a downward trend. The AAPC of ASIR, ASPR, and ASYR during this period were −0.61 (−0.66, −0.57), −0.66 (−0.71, −0.61), and −0.68 (−0.73, −0.64), respectively. This downward trend was particularly pronounced between 1990 and 1994, and as can be seen from the figure, this decline was mainly driven by a decrease among females. In terms of absolute numbers, however, there was an upward trend (Figures 6, 8).

**Figure 9:**
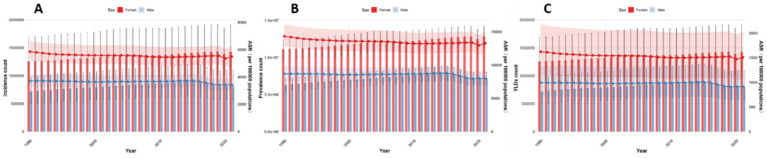
Number and rates of low back pain in Japan from 1990 to 2021: **(A)** Incidence, **(B)** Prevalence, **(C)** YLDs.

In Japan, the disease burden of low back pain is significantly higher than in other countries, yet it also exhibits a declining trend, with a more rapid decrease after 2015, primarily driven by a reduction in the burden among males. During this period, the AAPC of ASIR, ASPR, and ASYR were −0.28 (−0.36, −0.19), −0.30 (−0.36, −0.25), and −0.30 (−0.35, −0.24), respectively. In terms of absolute numbers, there was a slow upward trend among females, while among males, a downward trend was observed after 2015 (Figures 6, 9).

**Figure 10:**
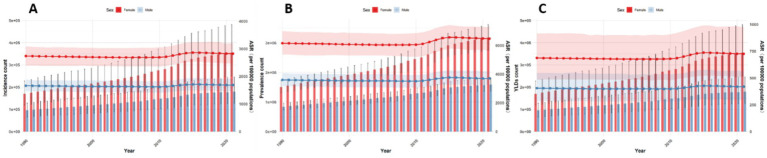
Number and rates of low back pain in Thailand from 1990 to 2021: **(A)** Incidence, **(B)** Prevalence, **(C)** YLDs.

In Thailand, the disease burden of low back pain is significantly lower than in other countries, but it exhibited an increasing trend between 2010 and 2015. The AAPC of ASIR, ASPR, and ASYR were 0.08 (0.07, 0.09), 0.14 (0.12, 0.16), and 0.16 (0.14, 0.18), respectively. Overall, the trends were similar for both males and females, but the increase was more pronounced among females during the 2010–2015 period. In terms of absolute numbers, both males and females experienced an upward trend, with a more significant increase observed among females (Figures 6, 10).

**Figure 11:**
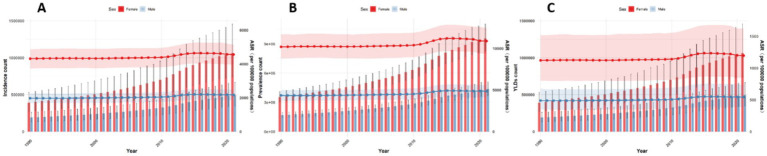
Number and rates of low back pain in Pakistan from 1990 to 2021. **(A)** Incidence, **(B)** prevalence, **(C)** YLDs.

In Pakistan, the level of disease burden is comparable to that of China, but it shows a more pronounced upward trend, especially with a significant increase in incidence and YLDs around 2010. The trend began to decline around 2017. The AAPC of ASIR, ASPR, and ASYR were 0.28 (0.26, 0.30), 0.32 (0.27, 0.36), and 0.32 (0.29, 0.36), respectively. In terms of absolute numbers, there was a more substantial upward trend compared to the rates, with a particularly significant increase observed among females (Figures 6, 11).

### Forecasting the burden of low back pain in global, China, Japan, Thailand, and Pakistan from 2022 to 2050 using the BAPC model

**Figure 12:**
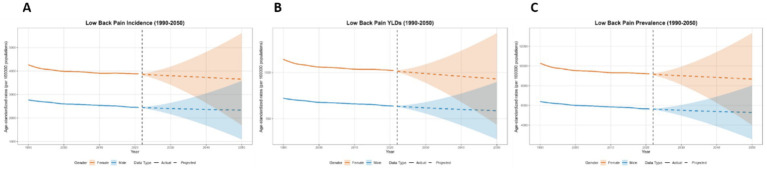
Predicted age-standardized rates of low back pain regarding the sex globally from 1990 to 2050: **(A)** Incidence, **(B)** Prevalence, **(C)** YLDs.

**Figure 13:**
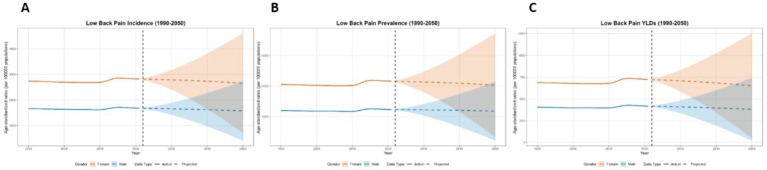
Predicted age-standardized rates of low back pain regarding the sex in China from 1990 to 2050: **(A)** Incidence, **(B)** Prevalence, **(C)** YLDs.

**Figure 14:**
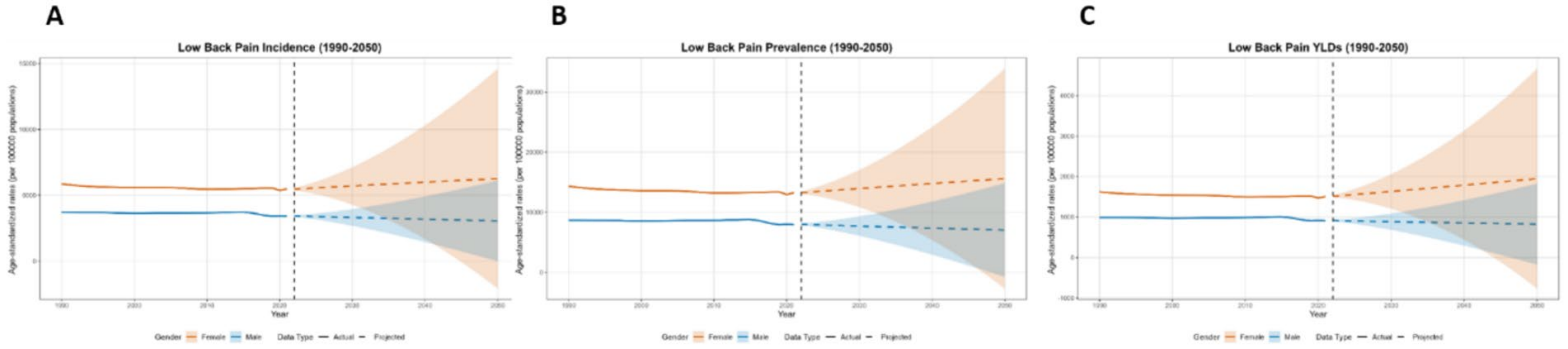
Predicted age-standardized rates of low back pain regarding the sex in Japan from 1990 to 2050: **(A)** Incidence, **(B)** Prevalence, **(C)** YLDs.

**Figure 15:**
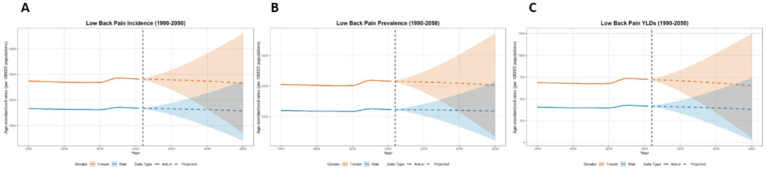
Predicted age-standardized rates of low back pain regarding the sex in Thailand from 1990 to 2050: **(A)** Incidence, **(B)** Prevalence, **(C)** YLDs.

**Figure 16:**
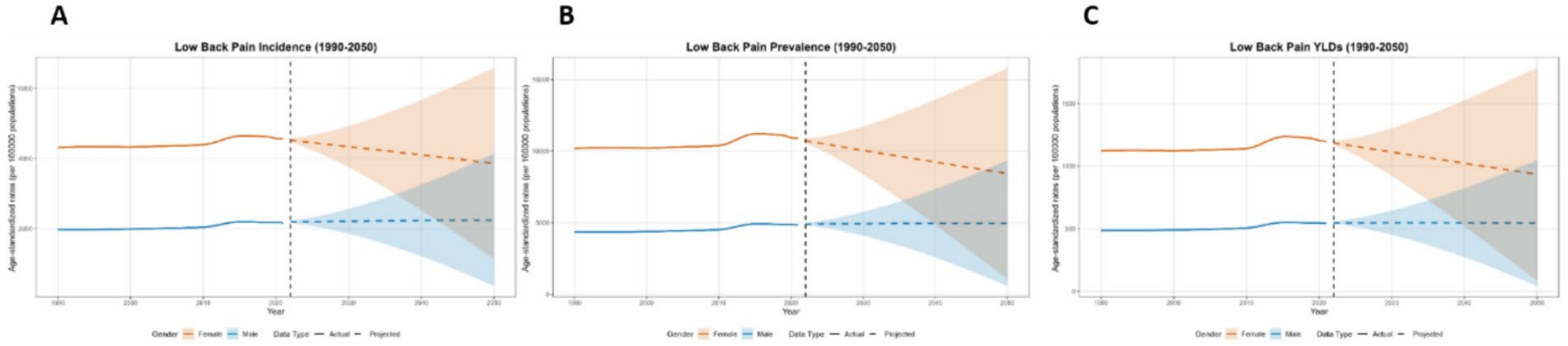
Predicted age-standardized rates of low back pain regarding the sex in Pakistan from 1990 to 2050: **(A)** Incidence, **(B)** Prevalence, **(C)** YLDs.

This paper employs the BAPC model to forecast the disease burden of low back pain from 2021 to 2050 in Global, China, Japan, Thailand, and Pakistan. The key projected values are summarized in Table 2, with the main findings below. At the global level, a general decline in disease burden is projected by 2050 for both sexes (Figure 12). The most notable decrease is seen in the ASYR for females (−8.95%). Diverging trends are observed between sexes in China and Japan (Figures 13, 14). In China, theASIR is projected to decrease by 3.37% for females but increase by 5.08% for males. Conversely, Japan is projected to see a substantial increase in the female ASYR (+29.40%) alongside a decrease in the male ASIR (−10.49%). Thailand’s pattern is similar to the global trend, with projected decreases for both sexes, such as a − 10.03% change in female ASYR (Figure 15). In Pakistan, the female disease burden is projected to decline significantly (e.g., ASIR: −15.35%), while male burden remains relatively stable with a minimal change in ASYR (+0.18%) (Figure 16).

**Table 2:** Burden of low back pain in China, Japan, Thailand, and Pakistan: 2021 and projected changes to 2050.

Location	Sex	ASIR (per 100,000)			ASPR (per 100,000)			ASYR (per 100,000)		
		2021	2050 projection (Mean ± 1.96SD)	% Change	2021	2050 projection (Mean ± 1.96SD)	% Change	2021	2050 projection (Mean ± 1.96SD)	% Change
Global	Female	3879.94	3652.30 ± 1959.63	−5.87%	9212.46	8668.70 ± 4652.92	−5.90	1021.52	930.07 ± 496.86	−8.95
	Male	2450.54	2337.24 ± 1243.17	−4.62%	5640.22	5289.06 ± 2725.52	6.23	635.48	586.53 ± 311.23	−7.70
China	Female	2779.15	2685.61 ± 2830.87	−3.37%	6381.37	6119.71 ± 6973.56	−4.10	716.14	673.23 ± 783.22	−5.99
	Male	1901.61	1998.17 ± 1877.01	+5.08%	4282.29	4577.00 ± 4314.74	6.88	488.34	521.42 ± 490.86	6.77
Japan	Female	5486.58	6257.22 ± 8349.45	+14.05%	13274.94	15592.12 ± 18280.57	17.46	1506.13	1948.87 ± 2713.35	29.40
	Male	3405.64	3048.43 ± 3060.92	−10.49%	7954.96	7047.96 ± 7774.28	−11.40	909.51	826.47 ± 998.33	−9.13
Thailand	Female	2822.96	2656.50 ± 1944.39	−5.90%	6473.17	6092.74 ± 5068.05	−5.88	724.47	651.81 ± 598.82	−10.03
	Male	1682.71	1574.60 ± 1161.01	−6.42%	3703.67	3537.37 ± 2886.87	−4.49	419.13	381.70 ± 358.73	−8.93
Pakistan	Female	4559.42	3859.54 ± 2716.21	−15.35%	10886.31	10886.31	−22.47	1197.06	934.59 ± 848.29	−21.93
	Male	2171.62	2248.46 ± 1882.83	+3.54%	4850.22	4965.00 ± 4391.08	2.37	543.88	544.84 ± 502.94	0.18

### Analysis of changes in low back pain disease burden in global, China, Japan, Thailand, and Pakistan from 1990 to 2021 using decomposition analysis

**Figure 17:**
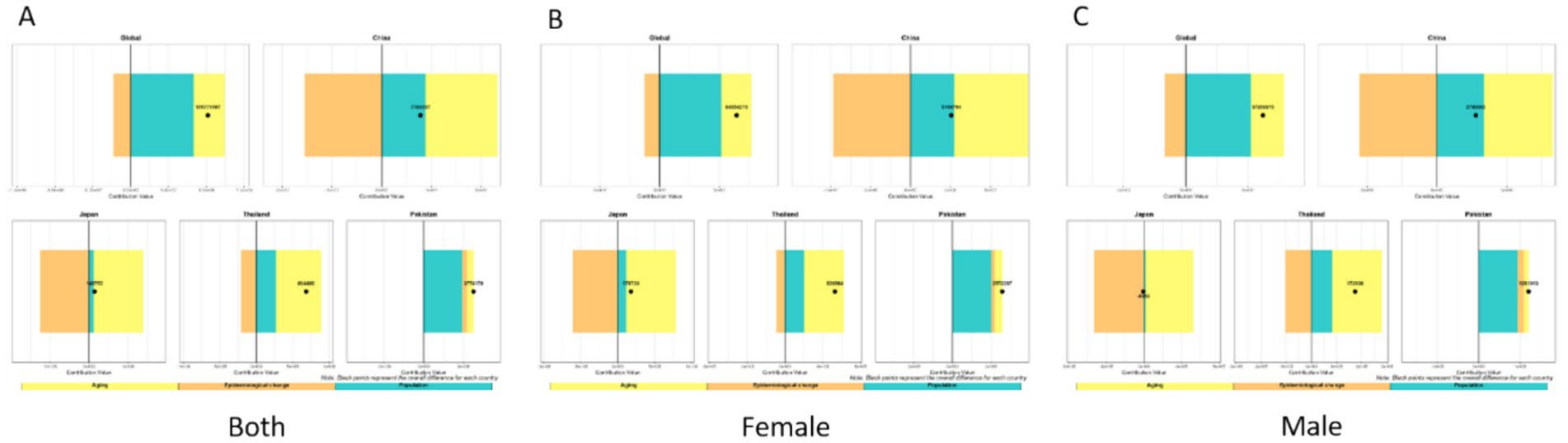
Decomposition method of low back pain incidence from 1990 to 2021 **(A–C)**. Both sexes **(A)**, females only **(B)**, males only **(C)**.

We analyzed the burden of low back pain across three metrics: incidence, prevalence, and YLDs. For incidence, the disease burden of low back pain is on the rise in all countries, but the increase is relatively smaller in Japan. Aging and population growth increased disease burden in both Global, China, Japan, and Thailand, while epidemiological changes decreased it. Notably, the contribution patterns varied: Globally, the increase was driven by population growth (81.7% contribution) and aging (41.0%), partially offset by epidemiological changes (−22.7%). In China, the increase was driven by aging (185.8%) and population growth (113.7%), which were largely offset by a strong negative contribution from epidemiological changes (−199.5%). In Japan, the small net increase resulted from a large positive contribution from aging (893.7%) being almost completely counteracted by a strong negative contribution from epidemiological changes (−876.1%), with a smaller positive contribution from population growth (82.5%). Conversely, Pakistan experienced burden increases from all three factors: population growth (77.3%), epidemiological changes (10.1%), and aging (12.6%). For Thailand, the increase was primarily driven by aging (90.8%) and population growth (39.5%), with a negative contribution from epidemiological changes (−30.3%). China and Japan demonstrated substantially larger increases from aging and reductions from epidemiological changes than Thailand or Global. The contribution patterns remained consistent between sexes within each country (Figure 17).

**Figure 18:**
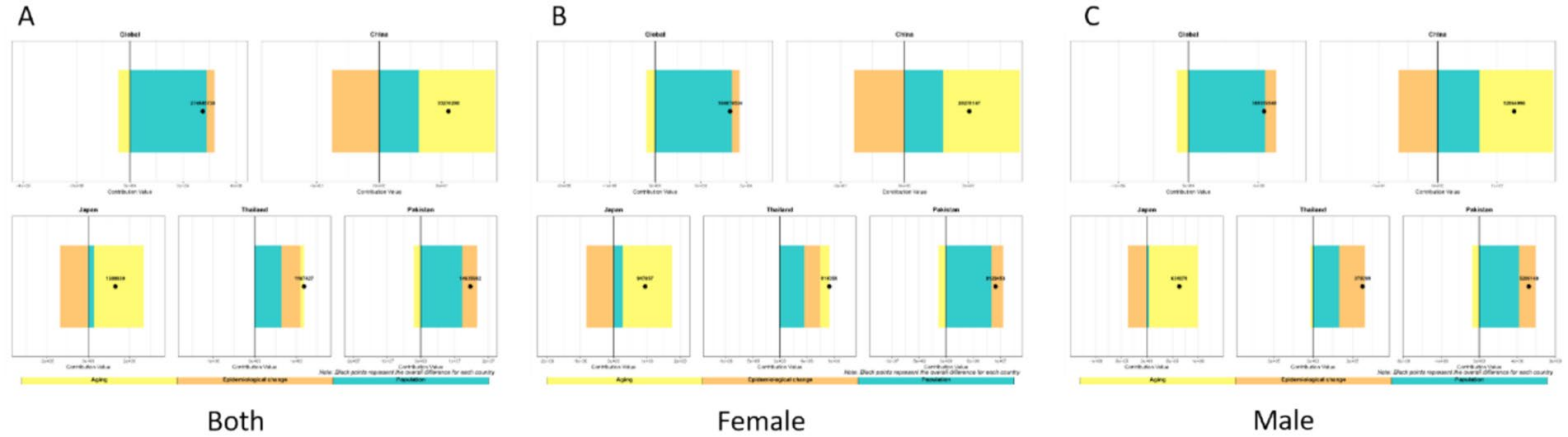
Decomposition method of low back pain prevalence from 1990 to 2021: **(A)** Both sexes, **(B)** females only, **(C)** males only.

Regarding prevalence, we found that the disease burden of low back pain is on the rise. Globally, the increase was driven primarily by population growth (104.9%) and, to a lesser extent, epidemiological changes (11.0%), which were partially offset by a negative contribution from aging (−16.0%). In Pakistan, population growth (83.7%) was the main driver, followed by epidemiological changes (30.1%), while aging had a negative contribution (−13.8%). In China and Japan, both aging (China: 110.5%; Japan: 183.4%) and population growth (China: 57.1%; Japan: 20.8%) were positive contributors. However, these were strongly offset by negative contributions from epidemiological changes (China: −67.6%; Japan: −104.2%), with the effect being particularly pronounced in Japan. For Thailand, all three factors demonstrated positive contributions: population growth (54.4%) was the primary driver, followed by epidemiological changes (38.5%) and aging (7.2%). Regarding sex differences, patterns were generally consistent between males and females across countries. However, in Thailand, population aging showed positive contributions in both the overall population and females specifically, but had a negative contribution among males (Figure 18).

**Figure 19:**
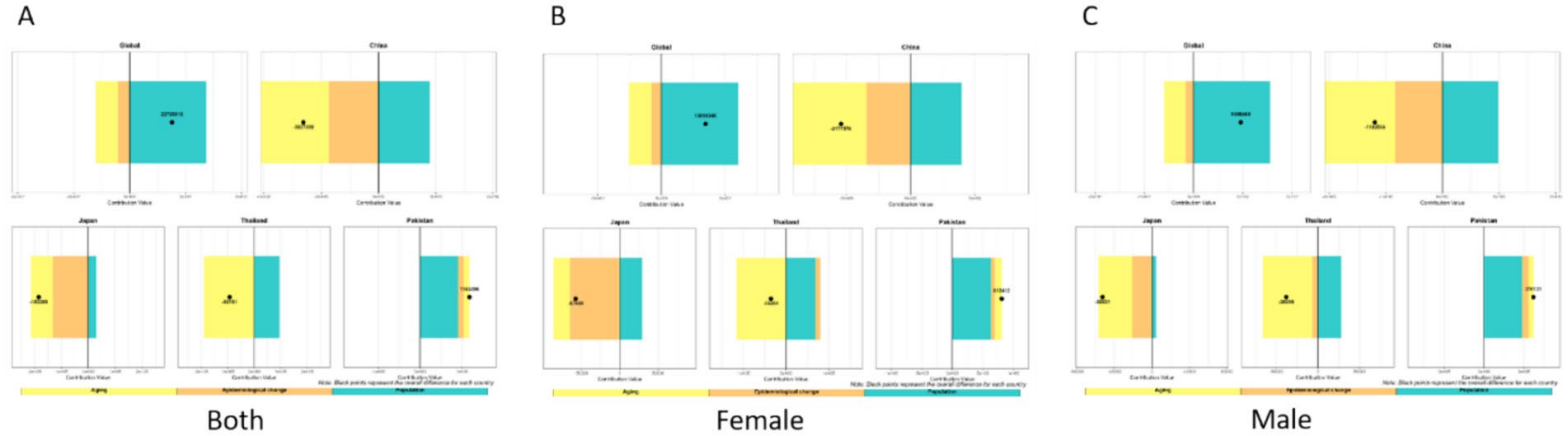
Decomposition method of low back pain YLDs from 1990 to 2021 **(A)** both sexes, **(B)** females only, **(C)** males only.

Regarding YLDs, overall low back pain burden decreased in China, Japan, and Thailand, but increased globally and in Pakistan. Our decomposition analysis revealed that: Globally, the increase was driven by population growth (180.2%), which was partially offset by negative contributions from aging (−52.3%) and epidemiological changes (−27.9%). China’s decrease was driven by strong negative contributions from both aging (−101.6%) and epidemiological changes (−66.2%), which outweighed the positive contribution from population growth (64.8%). Japan’s decrease was primarily due to a very strong negative contribution from epidemiological changes (−71.2%), alongside a negative contribution from aging (−45.0%). In Thailand, population growth was a positive contributor (104.5%) and population aging a strong negative contributor (−202.4%), while epidemiological changes demonstrated a negligible impact (−2.1%). Conversely, in Pakistan, all three factors acted as positive contributors: population growth (76.9%), aging (12.1%), and epidemiological changes (11.0%). Across all countries and territories examined, patterns of contribution were largely consistent between sexes (Figure 19).

## Discussion

Lower back pain ranked first in terms of YLDs burden in GBD 2021, accounting for 7.7% of all-cause YLDs and posing a significant public health challenge (8). This study comprehensively analyzes the epidemiological characteristics of LBP across representative Asia-Pacific countries (China, Japan, Thailand and Pakistan) and compares them with the Global Burden of Disease. Key findings are: Females consistently show a higher LBP burden across all studied nations. Japan demonstrated the highest burden metrics (ASIR, ASPR, ASYR), followed by Global and Pakistan, with China and Thailand exhibiting the lowest rates. The absolute number of cases (incidence, prevalence, YLDs) concentrated in middle-aged populations (e.g., 50–54 years globally), ASRs were highest among the older adult (75–84 years globally). From 1990 to 2021, ASRs decreased globally and in China and Japan but rose in Pakistan and Thailand; concurrently, absolute case counts increased everywhere except Japan. Projections for 2022–2050 indicate divergent future trends across countries, such as increasing male burden in China and rising female burden in Japan. Decomposition analysis revealing distinct national drivers; for example, population growth increased incidence everywhere, but its effect on prevalence and YLDs was counteracted by epidemiological changes in China and Japan.

Across all analyses presented in this paper, we consistently found a substantially higher disease burden among women compared to men, observed in all age groups, years, and countries of varying income levels. This disparity persists through 2050. However, decomposition analysis revealed largely similar key drivers of the disease burden for both sexes across countries. This gender difference aligns with findings from numerous previous studies (5, 15). It can be explained by both biological and socio-cultural factors. Biological factors include anatomical differences, hormonal levels, and gene expression (15, 16). The pelvic floor muscles are typically weaker in women, which can compromise spinal stability and lead to non-specific mechanical low back pain (17). Furthermore, MRI research indicates that estrogen deficiency plays an important role: while men are more prone to intervertebral disk degeneration than premenopausal women, postmenopausal women show a markedly greater tendency toward severe degeneration compared to both same-aged men and pre- or perimenopausal women (18). Pregnancy-related low back pain also contributes, with a prevalence as high as 63%, and can severely affect quality of life (19). Socio-culturally, differences in education levels, family roles, psychological factors, and income may contribute, as women often perform more back-straining tasks like childcare and household chores (15). Furthermore, studies indicate that women tend to seek medical care later when experiencing lower back pain, potentially leading to a longer symptom duration and increased burden (15). This also emphasizes the need to allocate more resources to women.

In addition to the aforementioned shared characteristics, it is essential to develop targeted strategies that account for the specific circumstances of each country. For instance, a cohort analysis of middle-aged and older adults in Japan by Takahashi et al. (20) identified older age and higher BMI as significant contributors to low back pain (LBP) in middle-aged and older women. Furthermore, compared to men, middle-aged women with metabolic syndrome are more susceptible to LBP (21). Another study in Japan revealed that among women, professionals/technicians and sales workers reported a significantly higher prevalence of LBP than clerical workers (22). These findings highlight the need for policies in Japan to place greater emphasis on obesity management and specific occupational groups within the female population. In contrast, research specifically examining the relationship between gender and LBP in China is relatively scarce. Available cross-sectional studies indicate that factors such as poor health status, a higher number of chronic diseases, poor sleep quality, alcohol consumption, depression, and employment in agricultural work are associated with LBP among individuals aged 45 and above in China (23). Consequently, policy measures should target these high-risk groups. Meanwhile, research from Thailand reports a notably high incidence of LBP (61%) among female rice farmers, underscoring the urgency for agricultural nations like Thailand to prioritize the health of this workforce (23). Similarly, a cohort study in Pakistan by Siddiqui et al. (24) found that 75% of the LBP population was overweight or obese, indicating a pressing need for policy initiatives in Pakistan to address LBP specifically in the context of obesity.

Regarding age, this study found that the age-specific rates for incidence, prevalence, and YLDs peaked consistently among older populations in different Asia-Pacific countries, exhibiting a unimodal pattern that rose to a peak and then gradually declined with increasing age. This aligns with the GBD findings by Ferreira et al., where the prevalence peak was around 85 years old (5). The actual case numbers, however, peaked at different ages: globally, in China, and Thailand, peaks occurred in middle age; in Japan, during both middle and old age; and in Pakistan, in young adulthood. This discrepancy is explained by variations in national population age structures, as evidenced by the GBD 2021 population data. For instance, in China, where the peak case number occurred in middle age, the population in 2021 was predominantly composed of middle-aged individuals, with the 45–64 age group constituting approximately 32.5% of the total population, compared to 13.6% for the population aged 65 and above. Conversely, in Japan, a country with one of the world’s most aged populations, the peak was observed in both middle and old age, reflecting its demographic structure where the 65 and above population accounts for over 29% of the total, vastly outnumbering the younger cohorts. In stark contrast, Pakistan, with a much younger demographic profile (over 60% of the population under the age of 30), exhibited a peak in young adulthood. This clear alignment between the peak age for case numbers and the dominant age group within each country’s population underscores the critical influence of demographic composition on the epidemiological pattern of the disease. This alignment demonstrates that the peak age for case numbers is strongly influenced by the relative size of different age cohorts in the population. However, current research focus on older adults is insufficient. Bibliometric analysis reveals that studies on working-age adults outnumbered those on older adults by nearly 12:1 from 1993 to 2023, with research on older adults particularly scarce in Asia (25). Furthermore, 53% of randomized clinical trials exclude individuals over 65 (26). Additionally, the research focus for older adults has shifted from surgery to physical activity, highlighting differences from the working-age population (25). Together, this underscores the necessity of dedicating resources to the treatment and research of low back pain in older adults.

In this study, the ASRs of low back pain incidence, prevalence, and YLDs were the highest in Japan, followed by the global average and Pakistan, with China and Thailand showing the lowest rates. This aligns with global research, where ASRs of low back pain DALYs were highest in high-SDI countries and lowest in middle-SDI countries (8). A key factor explaining this disparity is the substantial gap in healthcare accessibility, diagnosis, and reporting practices (27). High-SDI countries possess more comprehensive healthcare systems and robust administrative databases, enabling more complete case identification and formal recording (28), which elevates the calculated ASR. Conversely, in many low and middle income countries (LMICs), barriers to healthcare access lead to systematic under-ascertainment of cases (29), resulting in a lower reported ASR despite a potentially high true burden. This surveillance gap resolves the apparent paradox wherein high occupational risks in LMICs (e.g., 80–90% heavy manual labor (30)) do not yield the highest reported ASRs, as much of the resultant burden remains outside the formal surveillance framework. The divergent economic burden patterns—characterized by high direct medical costs in high-income settings (27) versus severe productivity losses and out-of-pocket expenses in LMICs (31)—further attest to this pattern of delayed and informal care in the latter. Finally, differences in socioeconomic and behavioral factors, such as sedentary lifestyles in high-SDI countries and compounding psychosocial stressors in LMICs (28), may also contribute to the observed burden profiles.

Regarding trends in disease burden: From 1990 to 2021, trends declined globally and in China and Japan, but increased in Thailand and Pakistan. Projections for 2022–2050 suggest declines globally and in Thailand and Pakistan, while China and Japan remain relatively stable. Notably, among all national projections, only Japanese females and Chinese males show increasing burdens, requiring focused attention. Addressing these trends requires targeted strategies: enhancing surveillance and research to identify driving factors, implementing tailored prevention and early intervention for these specific demographics, optimizing health systems to consolidate gains and address inequalities, and promoting a “health in all policies” approach to create supportive environments. This multi-layered response is crucial to counter rising risks in these groups and improve overall population health.

Our data, derived from the GBD 2021 model, were compared with relevant WHO estimates (32) to validate model accuracy. In that study, low back pain incidence was 22% in China and 39.1% in India (ages 50–70), while our estimates for the same age group in 2015 were 12% in China and 17.2% in Pakistan, suggesting possible underestimation in GBD. Conversely, a systematic review (33) in high-income countries reported incidence of 27–49% among those aged 60+, closer to our estimate for Japan (23.5%). Discrepancies may be due to differences in inclusion criteria or underreporting in low-SDI settings. However, due to limited national trend studies, we could not further validate trends externally. Uncertainty intervals (95% UI) were used to quantify data and modeling uncertainties; relatively narrow UIs for countries like Pakistan and Thailand suggest acceptable input data quality. Our decomposition analysis reveals that while population growth universally increased LBP burden, the diverging contributions of aging and, most critically, *epidemiological changes* shaped distinct national trajectories. Aging showed a positive contribution to incidence globally and in China, Japan, Pakistan, and Thailand, likely due to age-related spinal degeneration, osteoporosis, and comorbidities (1). The strong negative contribution of epidemiological changes to incidence, prevalence, and YLDs in China and Japan likely reflects effective public health interventions and healthcare improvements, which offset demographic pressures (28). Conversely, its positive contribution in Pakistan across all metrics suggests rising risks, potentially due to prevention gaps or healthcare access limitations (31). Thailand’s mixed pattern, particularly the increased burden among females, indicates gender-specific disparities. In response, differentiated strategies are warranted: China and Japan should sustain and disseminate their effective public health and occupational health strategies while enhancing chronic pain management for aging populations. Pakistan requires prioritized improvements in primary healthcare access, implementation of community-based prevention programs, and strengthening of pain diagnosis and treatment systems. Thailand needs gender-sensitive health interventions, with a particular focus on occupational health protection for female workers. Given that YLDs concentrate primarily in the working-age population (27), implementing these targeted measures is crucial not only for health equity but also for alleviating the overall socioeconomic burden of the disease.

**Figure 20:**
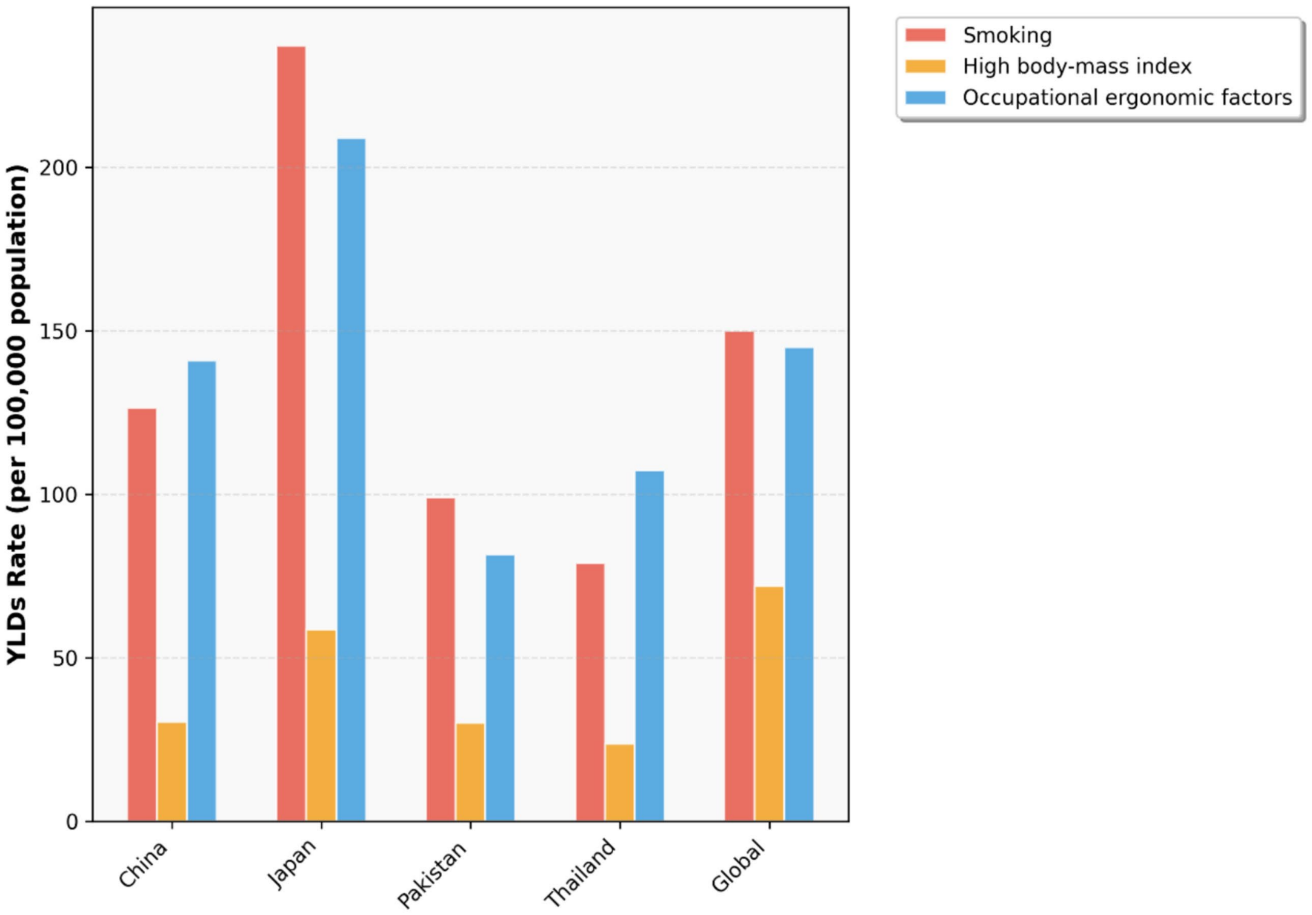
Age-standardized low back pain YLDs rate by risk factors, 1990.

**Figure 21:**
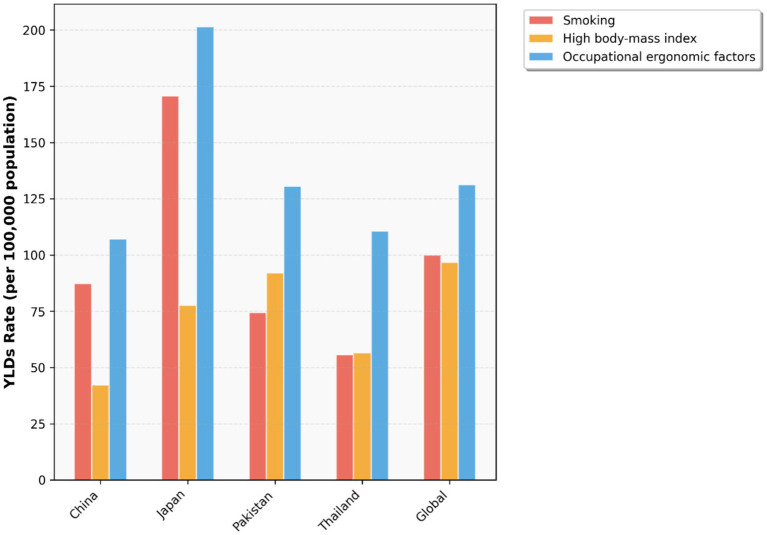
Age-standardized low back pain YLDs rate by risk factors, 2021.

**Figure 22:**
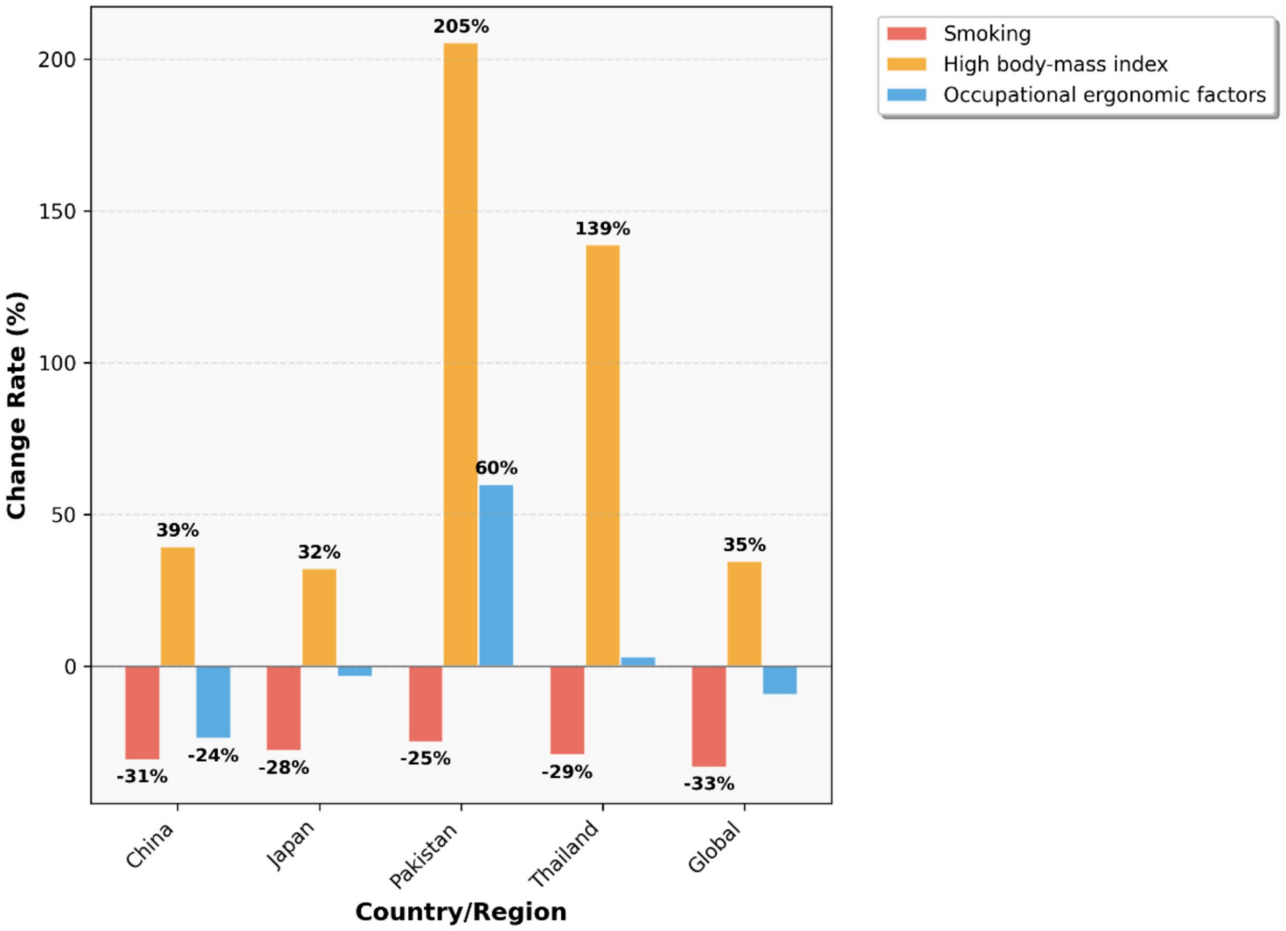
Low back pain YLDs by risk factor, 1990 vs. 2021.

To investigate the changes in various risk factors across different countries from 1990 to 2021 and provide a foundation for formulating relevant policies, we plotted Figures 20–22 using data from 1990 and 2021. These figures illustrate the variations in different risk factors over the period. Occupational ergonomic factors have now become the primary contributor to low back pain in all regions included in this study. Specifically, their contribution increased in Pakistan, slightly rose in Thailand, while decreasing in the other regions analyzed in this paper. The contribution of High body-mass index to low back pain significantly increased across all studied regions. Pakistan exhibited the largest increase in this factor, whereas Japan showed the smallest growth. In terms of Smoking, there was a substantial global decline in its contribution to low back pain. This trend indicates remarkable progress and positive outcomes of tobacco control efforts at the global level. In summary, our findings suggest that formulating policies targeting high BMI in the Asia Pacific region, as well as developing strategies addressing Occupational ergonomic factors in low-income countries such as Pakistan, will be highly effective in preventing and controlling low back pain.

Based on the established evidence in the literature, and while acknowledging that the present study was not designed to directly examine these specific risk factors, future research should prioritize the following directions. Population-based studies demonstrate that low back pain exhibits distinct burden patterns, with occupation and obesity identified as primary risk factors (34). In LMICs, where a large portion of the population is engaged in agriculture, approximately 52% of agricultural workers experienced low back pain in the past year (29), yet specific protective policies for this group are often lacking (35). Concurrently, high BMI has become a major and growing contributor to the global burden. Issues stemming from high BMI nearly doubled between 1990 and 2021, with a rapid rise in LMICs, though high-SDI countries also bear a significant load; for example, in the U. S., high BMI accounted for 11.4% of low back pain causes in 2021, with the burden among women nearly twice that of men (28, 36). Globally, DALYs attributable to high BMI-related low back pain increased by 170.97% from 1990 to 2021, a trend projected to continue (36). Given that our analysis did not assess these specific associations, future work is needed to address these evidence gaps. Key directions should include investigating the mechanisms and preventative strategies for occupation-related low back pain among manual laborers in LMICs, and conducting targeted longitudinal and interventional studies to mitigate the rising burden linked to high BMI, particularly among women and in populations undergoing rapid nutritional transition.

This study is subject to several limitations. The primary limitation is the considerable variation in the definitions of low back pain and recall periods used in data collection across different countries. Although the GBD employs regression methods to improve the comparability of data from various sources, these methods rely on a limited number of reliable studies and may introduce additional uncertainties (5). In low-income countries like Pakistan, issues such as insufficient coverage of epidemiological surveillance systems, limited diagnostic capabilities, and incomplete reporting from primary healthcare facilities may also contribute to data gaps (37). Conversely, for countries with effective prevention strategies such as China, GBD data might overestimate the disease burden, potentially leading to significant overestimation (38). Furthermore, it is important to note that our study includes only four countries. While they are broadly representative, the absence of certain key nations—such as India, a major lower-middle-income country, and Australia, a high-SDI comparator—limits the generalizability of the findings. Additionally, this ecological analysis did not account for individual-level risk factors (such as specific occupational exposures, BMI trends, or psychosocial determinants) that influence the burden of low back pain; their roles and interactions remain to be elucidated in future studies. Future studies could include these countries to enhance generalizability and further clarify the relationship between SDI and disease burden.

In summary, this study delineates the characteristics of lower back pain disease burden trends from 1990 to 2021 across four representative Asia-Pacific countries, projects its evolution from 2022 to 2050, and benchmarks these patterns against global standards. This analysis yields country-specific burden profiles to inform policy development and represents the first comparative study of its kind among Asia-Pacific nations, simultaneously underscoring the imperative for enhanced regional collaboration.

## Data Availability

The original contributions presented in the study are included in the article/Supplementary material, further inquiries can be directed to the corresponding author/s.
