# Supplementary material for: The burden of low back pain and predictions in Asia–Pacific region, 1990–2021: a comparative analysis of China, Japan, Thailand, and Pakistan
Source: Front Med (Lausanne). 2026 Feb 4;13:1693067. doi: 10.3389/fmed.2026.1693067 (PMC12913483; doi:10.3389/fmed.2026.1693067)
Supplement: Supplementary file 1 [file Table_1.DOCX]

**Bayesian Age-Period-Cohort (BAPC) Modeling for Low Back Pain Burden Projection**

**1. BAPC Model Specifications**

**1.1 Core Model Framework**

This study implemented the Bayesian Age-Period-Cohort (BAPC) model using the BAPC package (version 0.0.36) in R software (version 4.4.1)—a specialized package for Bayesian age-period-cohort models with a primary focus on projections. The BAPC package leverages integrated nested Laplace approximations (INLA) for full Bayesian inference, eliminating the need for traditional Markov Chain Monte Carlo (MCMC) methods. It generates both age-specific and age-standardized projected rates, and automatically incorporates Poisson noise when estimating the predictive distribution—critical for capturing the inherent variability of count data (like incidence cases of low back pain) derived from the Global Burden of Disease (GBD) 2021 database.

To account for population size differences across age groups, periods, and cohorts, the model incorporated population size as an offset term. The linear predictor of the model is defined as follows:

$$\boldsymbol{log}\left( \boldsymbol{\lambda}_{\left\{ \boldsymbol{ijk} \right\}} \right)\boldsymbol{=}\boldsymbol{\beta}_{\boldsymbol{0}}\boldsymbol{+}\boldsymbol{\alpha}_{\boldsymbol{i}}\boldsymbol{+}\boldsymbol{\pi}_{\boldsymbol{j}}\boldsymbol{+}\boldsymbol{\gamma}_{\boldsymbol{k}}\boldsymbol{+ log}\left( \boldsymbol{N}_{\left\{ \boldsymbol{ijk} \right\}} \right)$$

Where:

- $\boldsymbol{\lambda}_{\left\{ \boldsymbol{ijk} \right\}}$ denotes the expected incidence count of low back pain for the i-th age group, j-th period, and k-th cohort;
- $\boldsymbol{\beta}_{\boldsymbol{0}}$is the global intercept (baseline incidence level);
- $\boldsymbol{\alpha}_{\boldsymbol{i}}$epresents the age effect for the i-th age group (20 age groups: <5 years, 5-9 years, 10-14 years, ..., 95+ years);
- $\boldsymbol{\pi}_{\boldsymbol{j}}$enotes the period effect for the j-th time period (study period: 1990–2050, with 1990–2021 as observed periods and 2022–2050 as projected periods);

- $\boldsymbol{\gamma}_{\boldsymbol{k}}$ is the cohort effect for the k-th birth cohort (derived from age and period groups as cohort = period - age);
- $\boldsymbol{N}_{\left\{ \boldsymbol{ijk} \right\}}$ is the population size of the i-th age group, j-th period, and k-th cohort (offset term to adjust for population differences).

**1.2 Prior Distributions**

All model parameters adopted the **default weakly informative Gamma priors** of the BAPC package (version 0.0.36), which are specifically designed for the scale parameters of random effects in APC models, minimizing subjective constraints and allowing observed data to dominate parameter estimation. This is consistent with best practices for Bayesian APC modeling using the BAPC package:

- **Intercept**$\left( \boldsymbol{\beta}_{\boldsymbol{0}} \right)$: A flat (non-informative) prior was used, with a constant probability density across the entire real number line (**p(**$\boldsymbol{\beta}_{\boldsymbol{0}}$**) ∝ 1**), ensuring no subjective bias in the estimation of the baseline incidence level.
- **Age, period, and cohort effects** $\boldsymbol{(}\boldsymbol{\alpha}_{\boldsymbol{i}}$**,** $\boldsymbol{\pi}_{\boldsymbol{j}}$**,** $\boldsymbol{\gamma}_{\boldsymbol{k}}$**)**: First-order Random Walk (RW1) priors were specified by setting secondDiff = FALSE in the model call. This prior imposes smoothness on trends, which is critical for obtaining biologically and epidemiologically plausible results. For each effect, the first-order differences between adjacent levels follow a normal distribution:
- Age effect differences: Δ$\boldsymbol{\alpha}_{\boldsymbol{i}}$=$\boldsymbol{\alpha}_{\boldsymbol{i}}$−$\boldsymbol{\alpha}_{\boldsymbol{i-1}}$**∼N(0,*τ_α_*^2^​)**
- Period effect differences: Δ$\boldsymbol{\pi}_{\boldsymbol{j}}$=$\boldsymbol{\pi}_{\boldsymbol{j}}$−$\boldsymbol{\pi}_{\boldsymbol{j-1}}$​**∼N(0,*τ_π_*^2^​)**
- Cohort effect differences:  Δ$\boldsymbol{\gamma}_{\boldsymbol{k}}$​=$\boldsymbol{\gamma}_{\boldsymbol{k}}$−$\boldsymbol{\gamma}_{\boldsymbol{k-1}}$​**∼N(0,*τ_γ_*^2^​)**
- Where***τ_α_***, ***τ_π_***, and ***τ_γ_*** are scale parameters controlling the variability of age, period, and cohort effects, respectively. Each scale parameter was assigned the default Gamma prior of the BAPC package:

***τ_α_***, ***τ_π_***,***τ_γ_*** ∼Gamma((1, 0.00005).

This weakly informative prior allows moderate variability in effects while avoiding excessive shrinkage of trends.

- **Residuals**: No additional prior was required for residuals, as the Poisson likelihood inherently characterizes the error structure of count data $\boldsymbol{(}\boldsymbol{y}_{\left\{ \boldsymbol{ijk} \right\}}\sim\left\{ \boldsymbol{Poisson} \right\}\left( \boldsymbol{\lambda}_{\left\{ \boldsymbol{ijk} \right\}}\boldsymbol{} \right)\boldsymbol{)}$ with Poisson noise automatically integrated into the predictive distribution by the BAPC package.

**1.3 Model Fitting and Projection Settings**

- **Input data preparation**: Observed incidence counts (1990–2021) and population data (1990–2021 observed + 2022–2050 projected) were structured into an APCList object with a 5-year interval (gf = 5).
- **Projection configuration**: The model was set to project low back pain burden for 29 periods (2022–2050) using predict = list(npredict = 29, retro = TRUE), with the BAPC package generating both age-specific and age-standardized projected rates.
- **Age standardization**: The GBD 2021 world population age standard was incorporated as the reference structure via the stdweight parameter, with weights calculated based on the standard population distribution (detailed in Section 2).

**2. Application of the GBD 2021 World Population Standard**

To ensure cross-sectional and temporal comparability of incidence rates across different populations and time periods, the **GBD 2021 world population age standard** was used to calculate age-standardized incidence rates (ASIRs), following these rigorous steps:

1. **Acquisition of standard population data**: Age-specific counts of the GBD 2021 world population standard were extracted from the official GBD 2021 database. The standard population covers 20 age groups that are consistent with the study’s age stratification (<5 years, 5-9 years, 10-14 years, ..., 95+ years), ensuring no misalignment in age grouping.
2. **Weight normalization**: Age-specific standard population counts were converted to proportional weights by dividing each age group’s count by the total standard population size. This normalization ensures the sum of weights equals 1, which is a prerequisite for valid age standardization.
3. **Model integration**: Normalized weights were passed to the stdweight argument in the BAPC() function. The model used these weights to compute age-standardized rates during both the estimation (1990–2021) and projection (2022–2050) phases. Specifically, the age-specific incidence rates were weighted by the corresponding standard population proportions, then summed to obtain the age-standardized rate.
4. **Consistency validation**: The consistency between the standard population age groups and the study’s age stratification was verified through cross-checking. No discrepancies were found, ensuring that age-standardized results are comparable across countries with different demographic structures.

**3. Model Convergence Diagnostics**

Since the BAPC package relies on Integrated Nested Laplace Approximations (INLA) for full Bayesian inference rather than traditional Markov Chain Monte Carlo (MCMC) methods, the R-hat statistic (which depends on variance comparisons across multiple chains) is not applicable for convergence diagnosis. To ensure the reliability of model inference, we adopted **INLA-specific convergence validation strategies** as follows:

1. **Stability of the marginal log-likelihood (MLL):**​ We confirmed that the MLL, a key output of the INLA algorithm reflecting the model's overall fit, was stable and showed no anomalous fluctuations, indicating that the numerical approximations were successful.
2. **Examination of hyperparameter posteriors:**​ The posterior distributions of the precision parameters for the random walk effects were examined. Their distributions were unimodal and concentrated within reasonable ranges, showing no signs of pathological behavior, which suggests the smoothness assumptions are appropriate and supported by the data.
3. **Posterior predictive checks (PPC):**​ We generated samples from the posterior predictive distribution and compared them graphically to the observed data. The predicted trends in age-specific and period-specific incidence rates showed good visual agreement with the observed trends, indicating that the model adequately captures the underlying structure of the data.

Additionally, visual inspection of observed vs. predicted value scatter plots and age-specific/period-specific trend comparisons showed a high degree of overlap between observed and predicted data. These results confirm that the model adequately captures the underlying patterns in the observed data.

All three diagnostic indicators demonstrate that the model converged well, and the approximate inference results are reliable for subsequent interpretation and projection.

**4. Software and Packages**

All analyses were performed in R software (version 4.4.1). The following R packages were used:

- BAPC (v0.0.36): Specialized for Bayesian age-period-cohort model fitting and projection, generating age-specific/age-standardized projected rates and integrating Poisson noise for predictive distributions.
- INLA (v24.12.11): Provides full Bayesian inference via integrated nested Laplace approximations; installed using install.packages("INLA", repos="http://www.math.ntnu.no/inla/R/testing") (available from [http://www.r-inla.org](http://www.r-inla.org/)).

1. Muff S, Signer J, Fieberg J. Accounting for individual-specific variation in habitat-selection studies: Efficient estimation of mixed-effects models using Bayesian or frequentist computation. J Anim Ecol. 2020 Jan;89(1):80-92. doi: 10.1111/1365-2656.13087. Epub 2019 Sep 9. PMID: 31454066.

2. Boonstra PS, Barbaro RP, Sen A. Default Priors for the Intercept Parameter in Logistic Regressions. Comput Stat Data Anal. 2019 May;133:245-256. doi: 10.1016/j.csda.2018.10.014. Epub 2018 Nov 5. PMID: 31530966; PMCID: PMC6748335.
